# Investigating the Relationship between Stress and Self-Rated Health during the Financial Crisis and Recession in 2008: The Mediating Role of Job Satisfaction and Social Support in Spain

**DOI:** 10.3390/jcm10071463

**Published:** 2021-04-02

**Authors:** Raquel Sánchez-Recio, Cristina García-Ael, Gabriela Topa

**Affiliations:** 1 Department of Preventive Medicine and Public Health, Faculty of Medicine, University of Zaragoza, 50009 Zaragoza, Spain; rzanchez@unizar.es; 2 Faculty of Psychology, National Distance University (UNED), 28046 Madrid, Spain; gtopa@psi.uned.es

**Keywords:** mediation, work-related Stress, self-rated health, social support, job satisfaction and economic recession

## Abstract

Background: the 2008 financial crisis and subsequent recession had a strong impact on employment and certain health indicators, such as mental health. Many studies carried out with diverse samples attest to the negative influence of stress on health. However, few studies focus on stress and self-rated health among the Spanish workforce, or analyse which variables can act as a buffer against the negative effects of stress on self-perceived health. Aim: to analyse the mediator role of social support and job satisfaction in the relationship between work-related stress and self-rated health among the Spanish working population between 2006 and 2017. Method: repeated cross-sectional study using Spanish Surveys from 2006 to 2017, a total of 32.105 participants (47.4% women) aged 16 years and over (*M* = 42.3, *SD* = 10.7) answered a series of questions about work-related stress (PV), self-rated health (CV), job satisfaction, and social support (mediator variables) through the National Health Survey (NHS) prevalences of work-related stress, self-rated health, job satisfaction, and social support were calculated (standardised by age). We performed mediation/moderation analysis with Macro Process for SPSS to analyse the role of social support and job satisfaction in the relationship between self-rated health and work-related stress among the Spanish working population. Results: three mediation analyses were conducted, one for each time point in the study period. The results revealed a significant direct association between stress and job satisfaction. In the 2006 model, both job satisfaction and social support acted as mediators between stress and self-rated health, while in the 2011 and 2017 models, only job satisfaction acted as a mediator. The data reveal that the working population in Spain has a good capacity for resilience, since no drop in health indicators was observed. Conclusion: following the economic recession, employment has partially recovered. However, social and employment policies are required to help the population face the recent situation triggered by the Coronavirus crisis.

## 1. Introduction

Since 2008, the European Union (EU) has undergone one of the most severe economic recessions of its history. Numerous countries have experienced a drastic drop in their gross domestic product (GDP), coupled with an increase in public debt and more expensive loans [1,2,3,4,5,6]. At an individual level, many people have had their financial solvency threatened by job loss, a drop in salary, or reduced public spending on social welfare [7]. One of the main characteristics of this recession was the considerable rise in unemployment and job insecurity rates, mainly among the youngest and oldest members of the workforce [8]. Spain is one of the countries where the economic crisis has had the greatest impact. Although the crisis began in 2008, it was not until the first quarter of 2009 that its first consequences became apparent, and direct and indirect effects of its impact on health can be differentiated [9,10]:

- The direct effects can be classified as: (i) influence on the social structure of the population: the economic crisis has influenced the welfare of the population by sharply decreasing employment, increasing poverty rates, and the lack of social policies. (ii) Influencing health systems to contain economic expenditure.
- The indirect effects: loss of priority in health for both governments and the population itself as economic problems take precedence.

It is very difficult to monitor the health consequences of the various social, health and employability measures taken during the economic crisis of 2008. Monitoring this fact, the effects of the crisis on the health of different groups of the population, is important in order to assess whether the basic criteria of equity and efficiency with regard to citizens’ health have been met.

The association between employment and health is well-known [11]. Many authors have described a protective effect of employment on self-rated health. Studies carried out in Spain during the economic crisis in this respect show divergent results depending on the methodology used. Some authors, such as Arroyo et al. [12] conclude that there are no statistically significant differences in the self-perceived health of the Spanish population before and during the economic crisis. However, Aguilar Palacio et al. [13] in a study carried out with data provided by the National Health Surveys in Spain, showed that women, after entering the labour market, reported improvements in their self-perceived health during this period of economic recession. Factors that may have a positive impact on this relationship include job satisfaction, social support, both inside and outside the organisation, financial compensation, and the possibility of developing one’s professional career. However, certain risk factors may have a negative effect. These factors include stress, job instability, precarious labour contracts, and the absence of prospects for advancement (in both financial and professional terms) [13,14]. Many previous studies focusing on the working population have demonstrated the effects of stress on certain health indicators, such as mental health. However, few studies have analysed the relationship between self-rated health and stress, or whether variables, such as social support or job satisfaction attenuate the negative effects of stress on self-rated health, and even fewer have been carried out with population-wide measures, such as the Spanish National Health Survey (NHS). Consequently, the main aim of the present study is to analyse the relationship between self-rated health and work-related stress among the working population in Spain from 2006 to 2017, as well as the mediating role of social support and job satisfaction in the previous mentioned relationship, in order to identify the possible impacts of the financial crisis of 2008 and subsequent recession.

### 1.1. Work-Related Stress

Stress is also understood as the organism’s reaction to situations of “danger”, designed to enable it to adapt to the new situation. In the work environment, a certain degree of stress is necessary to ensure optimal productivity levels. However, it has also been shown that when stress levels generate an imbalance in the organism, they are associated with a loss of both physical and psychological health [15,16]. 

From the perspective of organizational psychology, there are many theoretical approaches to stress. Nevertheless, the Job Demands–Resource (JD–R) model [17] offers an integrative theoretical approach focused on the relationships between stress antecedents and consequences. Starting from the basic premises of the Demand–Control (DC) model [18], the JD–R suggests that demands (associated with psychological or physical costs) and resources (associated with psychological or physical strengths).

There are several different risk factors that influence the presence of stress, including: (1) factors linked to the environment and the organisation, such as a general recession or the type of job in question [19]; (2) individual factors, such as seniority and degree of specialisation, personality, self-esteem, gender, and age, etc. [19]; and (3) the situation of the labour market and underemployment [20]. Inversely, protective factors include: (1) job satisfaction and/or work engagement [21]; (2) organisational support [22]; and (3) the social support received by the individual in question [23,24], among others. In this sense, a direct relationship has been found between lower levels of work-related stress, social support, job satisfaction, and psychological distancing [25]. In this vein, several studies have demonstrated that the interaction between work demands, social support, and job demands (no control) predict health symptoms, absence of disease, organizational commitment, and satisfaction with job supervisors [26], or that high levels of social support cause high levels of intrinsic motivation, regardless of demand and control levels [27].

There are also new emerging risk factors for work-related stress. One such factor is job insecurity, understood as the perceived inability to maintain job continuity in the event of a recession [24]. Job insecurity is a strong generator of work-related stress [28]. Lazarus and Folkman (1984) concluded that job insecurity poses a greater risk to an individual’s general wellbeing than actual job loss. In 2007, the European Agency for Safety and Health at Work identified work-related stress as the third most serious psychosocial risk, after precarious contracts in unstable jobs, and the increased vulnerability of workers in the context of globalisation [29].

### 1.2. Self-Rated Health

Self-rated health is a good multidimensional indicator of health that provides information about how a person feels physically and mentally at a given moment in time [30]. It has also been shown to help predict other indicators, such as morbidity and mortality, disability and even health service use [31,32,33].

The economic recession, which began in 2008, had a major impact on the health of the Spanish population, with those who lost their job or experienced a worsening in their financial circumstances being the hardest hit. One of the most powerful indicators in this respect is mental health. There are many studies in the scientific literature that report a worsening of mental health during this period [34,35]. However, in relation to self-rated health, the results found reveal that, despite what may be expected, the level of this indicator has not dropped among the Spanish population, and has even risen among certain groups, such as women with high qualification levels [12,36], link this finding to a shift in priorities, with employment and the economy taking precedence over health during periods of recession, although other authors have related the improvement in self-rated health observed among women with high qualification levels to an improvement in their employment status [37].

### 1.3. Social Support

Social support alludes to the feeling of being appreciated and valued by other people and forming part of a social network [36]. Thus, although the social relations derived from the current labour context may have a negative impact on health, the vast majority of studies suggest that a good social network (colleagues; managers; friends) is one of the most important and critical ways buffering the negative effect of work stress and, consequently, of promoting psychological well-being [12,38]. In fact, according to the JD–R model, social support could buffer the impact of job demands, considered as work- related stressors, on employee’s health [39].

Thus, research on the relationships between stress and social support (among others factors) has demonstrated that employees with relatively little social support at work or with jobs characterised by high psychological demands are at risk of developing worse mental health [40], whereas employees supported by leaders and co-workers feel less stressed are fairly rewarded for their efforts [41], and are better able and more effective to coping with stress [42]. In this vein, different studies have found that indicate that increased levels of perceived support can reduce the effects of stress on bad health during the coronavirus disease (COVID-19) pandemic [43]. Similarly, research suggests that high levels of social support (for medical staff) are positively related to self-efficacy and negatively to the degree of anxiety and stress [43].

### 1.4. Job Satisfaction

Job satisfaction is the positive emotional state linked to a positive assessment of one’s work-related experience [44]. It is a multi-causal construct influenced by: (a) individual characteristics such as age, gender, education level, work-related values, and family structure; and (b) characteristics linked to the job itself, such as salary, working hours, job security/insecurity, promotion prospects, interpersonal relationships, and relationships with superiors, with these last two being the most influential factors [45]. Job satisfaction is also directly associated with the presence of work-related stress and its repercussions on health. Several theories have been developed to help explain job satisfaction, with Herzberg’s Two Factor Theory (1967) and the Determinants of Job Satisfaction Model being particularly worth highlighting.

The Two Factor Theory [46] posits that job satisfaction and job dissatisfaction are two totally different elements. According to this theory, there are two types of need, which influence an individual’s perception of job satisfaction: (a) hygiene needs, such as the physical and psychological working environment; and (b) motivating needs, which are linked to the activities carried out within the job. If a worker’s hygiene needs are met, then even if they do not feel satisfied, they will not feel dissatisfied either. However, in order for a worker to feel satisfied, their motivating needs must also be met.

The Determinants of Job Satisfaction Model [44] focuses on the existence of an expectation-recompense relationship, in which the degree of job satisfaction depends on the recompense obtained through the activity performed, while dissatisfaction arises when this relationship is reversed. In this sense, a study carried out in Spain by Gamero (2005) found that job stability and prospects for promotion had a strong impact on job satisfaction. Furthermore, this study demonstrated that the factors that influenced job satisfaction most strongly were the activity carried out, job stability, the possibility of achieving a good work-life balance, financial recompense, and relationship with middle management [47].

### 1.5. Objective

In light of the above, the main aim of the present study is to analyse the relationship between self-rated health and work-related stress among the working population in Spain from 2006 to 2017, in order to identify the possible impacts of the economic recession which began in 2008. The study also aims to explore the possible protective effect of social support and job satisfaction on self-rated health, taking the possible existence of binary gender inequalities into consideration in all cases. Based on our review of the literature, the following hypotheses were formulated in accordance with the study’s general aims:

**Hypothesis** **1.** *Differences will be observed between men and women in relation to self-rated health, with women reporting poorer health.*

**Hypothesis** **2.** *A negative relationship will be found between work-related stress and self-rated health at the three time points analysed during the study period.*

**Hypothesis** **3.** *A positive relationship will be found between job satisfaction and self-rated health during the period analysed.*

**Hypothesis** **4.** *Social support and job satisfaction will mediate the relationship between work-related stress and self-rated health.*

## 2. Methodology

We used a repeated cross-sectional study design. This study was based on information provided by the National Health Surveys (NHSs) in 2006 [48], 2011 [49], and 2017 [50]. The NHSs are representative surveys with a stratified multistage design that are administered in the form of individual interviews [51]. To control for possible seasonal effects, all months of the year were taken into account during the sample selection. For further information about the surveys, see the references section [48,49,50]. They were performed by means of personal interview among a non-institutionalised population from 15 years old. Therefore, in order to homogenise the sample in this study, only those over 16 were included in the analysis. Seasonal effect was avoided by including autumnal months in the sample collection. The methodology applied allows comparability between surveys. Sample sizes ranged from 29,478 in 2006, 20,884 in 2011, and 22,903 in 2017.

In this study, we selected from these health surveys the “working population”. The term “working population” is used to refer to everyone aged 16 years and over who, at the time of the interview, had worked during the reference period for at least one hour, paid either in the form of monetary wages or in kind, including those who were off sick, on holiday or on any other kind of leave [52]. In Spain, the legal minimum age for employment is 16 years among emancipated minors. Those still living under the care of their parents or legal guardians require parental permission until age 18 [53]. Therefore, in this study, the participants were 32,105 members of the Spanish working population (47.4% women) aged 16 years and over (*M* = 42.3, *SD* = 10.7) during the study period (2006–2017).

### 2.1. Independent Variables

The independent variables included in the study were: (i) sociodemographic variables and mental health (descriptive variables): age: in the NHSs, age is recorded as a continuous quantitative variable; sex: in the NHSs, sex is a dummy variable (man/woman); social class: social class was extracted from the proposal made by the Working Group on Determinants of the Spanish Epidemiological Society (SEE), which assigns respondents a social class in accordance with their occupation; education level: this variable was calculated on the basis of the International Standard Classification of Education (ISCED) [54] and was re-coded into three categories: low (no education or primary education); medium (secondary education and vocational training); or high (university qualifications); mental health: in the NHSs, this variable is recorded using the General Health Questionnaire (GHQ) [55]. The questionnaire asks about 12 items linked to mental health over the previous two weeks. The Cronbach’s alpha obtained in the present study was 0.968.

### 2.2. Dependent Variables

Self-rated health: this was measured through a question, which aimed to measure respondents’ perceptions of their general health status over the last twelve months, specifically, the question used in national health surveys is: “how would you say your health has been in the last twelve months?” The question offered five response options: very good, good, regular, poor, and very poor health.

Work-related stress is measured in the NHSs through the following question: “Overall, and bearing in mind the conditions under which you work, how stressful would you say your job is?” Response options range from 1 (“Not at all Stressful”) to 7 (“Very Stressful”). For this study, the variable work stress was recoded into a dichotomous variable in which all respondents who answered 1 were considered to have no work stress, and the remaining responses (from 2 to 7) were grouped into the category of having work stress.

### 2.3. Mediator Variables

Job Satisfaction is measured in the NHSs through the following question: “Bearing in mind the characteristics of your job, how satisfactory would you say it is?” Response options range from 1 (“Not at all Satisfactory”) to 7 (“Very Satisfactory”).

Perceived Social Support was a synthetic variable generated through the functional social support questionnaire (Duke-UNC) questionnaire [56]. Given that this index was obtained using the NHS information gathering technique, to verify its reliability in our sample, we calculated the Cronbach’s alpha [57], obtaining a value of 0.968.

### 2.4. Data Analysis

First, a descriptive analysis of the sample was carried out in accordance with sociodemographic (age, social class, and education level) and health-related variables (mental health) (Hypothesis 1), as well as in terms of the principal study variables (criterion, predictor and mediator variables), for which correlations were also calculated. Trend analyses were conducted to study the evolution of self-rated health, work-related stress, job satisfaction and perceived social support across the study period (using Chi^2^ for categorical variables and Student’s *T* for continuous variables) (Hypothesis 2). Finally, to determine the mediator effect between self-rated health and work-related stress (Hypotheses 3 and 4), various regression analyses were carried out using the procedure designed by Baron and Kenny (1986). This procedure requires that the predictor (work-related stress), criterion (self-rated health) and mediator variables (job satisfaction and perceived social support) be positively correlated with each other. Due to the positive associations observed among self-rated health and age, sex, social class (in this case grouped into two categories: blue collar workers–social classes IV (qualified jobs), V (primary sector jobs), and VI (unqualified jobs), and white collar workers–social classes I (managers > 10 people), II (managers < 10 people), and III (middle management), and education level, we decided to control for the effects of these variables by entering them in the first step of the regression. To determine whether job satisfaction and perceived social support mediate the relationship between self-rated health and work-related stress, three mediation analyses were carried out using the Process macro for SPSS [58], one for each time point in the study period. Age (coded as 0 ≤ 40 years and 1 ≥ 40 years), sex (coded as 0 = men and 1 = women), education level (coded as 0 = non university and 1 = university), and profession (coded as 0 = unqualified jobs (social classes IV, V, and VI) and 1 = qualified jobs (social classes I, II, and III) were included as covariables. The bootstrapping technique with 10,000 subsamples was used to estimate the confidence interval (95%).

In all analyses, we applied the weighting factors provided by the NHSs to avoid errors linked to the design rather than to the response. Tests were considered significant when *p* < 0.05. The analyses were carried out using IBM SPSS Statistics 24^®^ and Stata 14^®^ (Zaragoza University licence). Since the microdata from the surveys are public and anonymous, we were not required to request ethical approval for this study.

## 3. Results

During the study period (2006–2017), the sociodemographic structure of the working population in Spain underwent certain characteristic changes (Table 1). These include a statistically significant drop in the number of men from social class III (25.1% vs. 20.5%, *p* = 0.016) and a statistically significant rise in the number of men from social class V (12.1% vs. 30.9, *p* < 0.001). There was also a statistically significant decrease in the number of women from social classes III (26.2% vs. 22.4%, *p* = 0.052) and IV (20.6% vs. 9.5%, *p* < 0.001), whereas the number of women from social class V rose, with the change also being statistically significant (12.1% vs. 30.9%, *p* < 0.001). In regards to education level, statistically significant changes were observed at all levels, with the number of people with medium and high qualification levels increasing, and the number of those with a low qualification level dropping.

**Figure 1:**
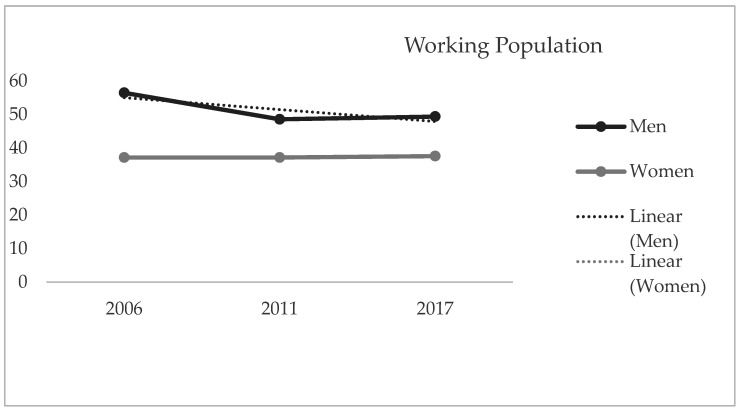
Working population (%) by year of study (2006–2017), disaggregated by sex.

As shown in Figure 1, a statistically significant drop was observed in the male working population (56.5% vs. 49.4%, *p* = 0.002). In terms of health-related characteristics, a statistically significant worsening was observed in the mental health of the Spanish working population (men: 4.8% vs. 9.1%, *p* < 0.001; women: 4.7% vs. 9.8%, *p* < 0.001). Nevertheless, the data pertaining to self-rated health revealed that a constant level was maintained throughout the study period among both men and women, with no statistically significant differences being found between the sexes (*p* = 0.240).

Table 2 shows the descriptive statistics and correlations between the criterion (self-rated health), predictor (work-related stress), and mediator variables (job satisfaction and perceived social support) included in this study. Across the three time points included in the study, the Spanish population reported good levels of self-rated health (scores of over 4). Work-related stress and job satisfaction followed similar trends throughout the entire study period, with scores above the mean. However, perceived social support dropped by almost 10 points, rendering the difference statistically significant (*p* < 0.001). The results therefore partially support Hypothesis 1, since while changes were observed in the social structure of the Spanish working population, along with a worsening in mental health, self-rated health levels did not drop, and no gender differences were found. The results also only partially support Hypothesis 2, since no changes were observed in work-related stress and job satisfaction levels among the Spanish working population, although perceived social support was found to decrease.

In relation to the bivariate correlation analyses carried out to determine the relationships existing between the principal variables in the study (criterion: self-rated health; predictor: work-related stress; and mediator: job satisfaction and perceived social support), in all cases, the assumptions of independence, collinearity, homoscedasticity, and linearity were met. As shown in Table 2, self-rated health correlated negatively and significantly with work-related stress (r_2006_ = −0.074; *p* < 0.001; r_2011_ = −0.079; *p* < 0.001; r_2017_ = −0.086; *p* < 0.001), and positively and significantly with job satisfaction (r_2006_ = 0.108; *p* < 0.001; r_2011_ = 0.126; *p* < 0.001; r_2017_ = 0.140; *p* < 0.001), and perceived social support (r_2006_ = 0.063; *p* < 0.001; r_2011_ = 0.078; *p* < 0.001; r_2017_ = 0.158; *p* < 0.001).

### Mediation Analyses

The percentage of the variance explained by job satisfaction in the relationship between work-related stress and self-rated health oscillated between 40% and 3.49%. In the case of perceived social support, the variance explained was 15.4% at the first time point and 4.98% at the third. Finally, in terms of the joint mediation of job satisfaction and perceived social support, the variance explained was 6.3% at the first time point and 18.38% at the third.

**Figure 2:**
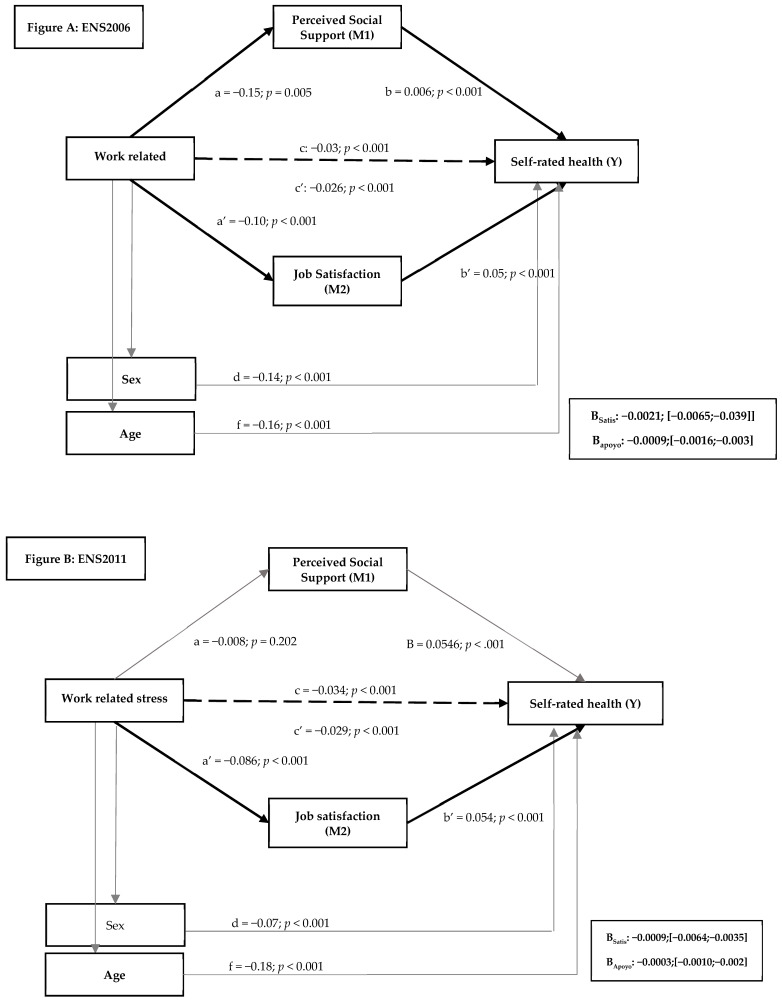

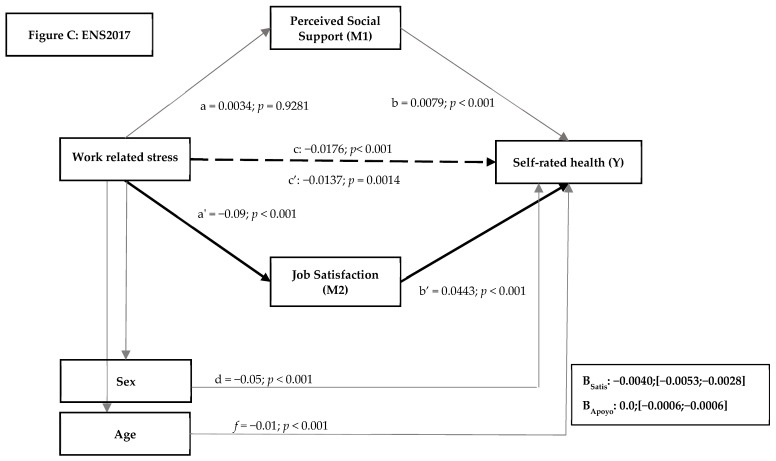
(**A**–**C**): Results of the mediation analysis. Criterion variable: self-perceived health, explanatory variable: job stress and mediating variables: job satisfaction and perceived social support, where a, b, c, a’, b’, c’ are the direct effects of medication. Indirect effects are represented by β_job satisfaction_ (indirect effect of satisfaction) and _βsocial support_ (indirect effect of perceived social support).

As shown in Figure 2A–C, the analyses revealed a direct significant effect between work-related stress and self-rated health at all three time points (β_2006_ = −0.0266, SE = 0.0038, *p* < 0.001, 95% CI (−0.0341, −0.0192); β_2011_ = −0.0297, SE = 0.0045, *p* < 0.001, 95% CI (−0.0385,−0.0208); β_2017_ = −0.0137, SE = 0.0043, *p* = 0.0014, 95% CI (−0.0221,−0.0053). Similarly, the direct effects of the mediator variables on self-rated health were significant at all three time points: job satisfaction (β_2006_ = 0.0516, SE = 0.0043, *p* < 0.001, 95% CI (0.0432, 0.0601); β_2011_ = 0.0546, SE = 0.0055, *p* < 0.001, 95% CI (0.0438,.0654); β_2017_ = 0.0443, SE = 0.0051, *p* < 0.001, 95% CI (0.0343, 0.0542)) and perceived social support (β_2006_ = 0.0059, SE = 0.006, *p* < 0.001, 95% CI (0.0047, 0.0071); β2011 = 0.0040, SE = 0.0007, *p* < 0.001, 95% CI (0.0025, 0.00549; β_2017_ = 0.0079, SE = 0.0012, *p* < 0.001, 95% CI (0.0056, 0.0101)).

In the analysis of the global model, the indirect effect of job satisfaction on self-rated health was significant at all time points (β_2006_ = −0.0051, SE = 0.0006, *p* < 0.001, 95% CI (−0.0065, −0.0039); β_2011_ = −0.0049, SE = 0.0007, *p* < 0.001, 95% CI (−0.0064, −0.0035); β_2017_ = −0.0040, SE = 0.0006, *p* < 0.001, 95% CI (−0.0053, −0.0028)). However, the indirect effect of perceived social support was only significant in 2006 (β_2006_ = −0.0009, SE = 0.0003, *p* < 0.001, 95% CI (−0.0016, −0.0003)). The covariables analysed were also found to influence the relationship between work-related stress and self-rated health. These results were applicable to men (β_2006_ = −0.14, SE = 0.0130, *p* < 0.001, 95% CI (−0.1643, −0.1143); β_2011_ = −0.0763, SE = 0.0156, *p* < 0.001, 95% CI (−0.1069, −0.0457); β_2017_ = −0.0697, SE = 0.0157, *p* < 0.001, 95% CI (−0.1005, −0.0389)), younger workers (β_2006_ = −0.16, SE = 0.0083, *p* < 0.001, 95% CI (−0.1778, −0.1453); β_2011_ = −0.1792, SE = 0.0102, *p* < 0.001, 95% CI (−0.1992, −0.1591); β_2017_ = −0.1780, SE = 0.0103, *p* < 0.001, 95% CI (−0.1952, −0.1578)), those with qualified jobs (β_2006_ = 0.221, SE = 0.0032, *p* < 0.001, 95% CI (0.158, 0.283); β_2011_ = 0.330, SE = 0.0408, *p* < 0.001, 95% CI (0.250, 0.410); β_2017_ = 0.276, SE = 0.037, *p* < 0.001, 95% CI (0.203, 0.3499) and those with a high education level (β_2006_ = 268., SE = 0.037, *p* < 0.001, 95% CI 80.194, 0.342); β_2011_ = 0.296, SE = 0.0308, *p* < 0.001, 95% CI (0.220, 0.337); β_2017_ = 0.169, SE = 0.037, *p* < 0.001, 95% CI (0.096, 0.349)).

These results indicate that job satisfaction directly mediated the relationship between work-related stress and self-rated health at all three time points included in the study. Perceived social support, on the other hand, was only found to mediate this relationship in 2006. We can therefore conclude that the results support Hypothesis 3, but only support Hypothesis 4 in relation to 2006.

## 4. Discussion

The aim of the present study was to analyse the relationship between self-rated health and work-related stress among the working population in Spain from 2006–2017, in order to identify the possible impacts of the economic recession, which began in 2008 and the effect of possible mediator variables.

The results reveal how, during the recession, the working population in Spain decreased, particularly in terms of the number of male workers. Likewise, our results also showed that work-related stress, self-rated health, and job satisfaction levels remained constant throughout the entire period (2006–2017), thereby indicating that they were not affected by the financial crisis and subsequent recession. We observed a negative association between work-related stress and self-rated health, and this relationship was found to be positively mediated by job satisfaction. However, we also observed that perceived social support only had the same positive mediating effect in the years prior to the recession (2006).

In relation to gender differences in job loss, our results are consistent with those described previously in the scientific literature [11,59]. In Spain, the recession, which began in 2008 was characterised by heavy job losses among blue-collar workers, with jobs linked to the social field and those most closely related to the female sector being impacted less severely [59]. This same pattern has been identified also in previous recessions [60,61]. The education level among the Spanish population increased, but this was not reflected in a consequent rise in social class, with the exception of women in social class V. There are several possible reasons for this. Spain was one of the countries in the European Union that was most directly impacted by the recession, with economic inequality rising sharply, mainly due to a lack of social and employment policies [62]. Another reason may have been the brain drain to other countries offering better job opportunities [63].

Other authors have reported how the recession and its consequent widespread job loss had a negative influence on the mental health of the Spanish population, a finding which is consistent with our results [35,36,64]. Furthermore, other studies have shown how the self-rated health of the Spanish people grew no worse during the recession [13,59], or even, according to Aguilar-Palacio et al. (2018), improved among women as they raised their employment status and took on the role of breadwinner [13].

In times of recession, job insecurity is known to be one of the principal sources of stress and the factor responsible for important alterations in individuals’ physical and mental health [10,11,14]. However, this stressor does not influence everyone equally. Those with a good social environment are better able to cope with these situations and suffer less severe health-related consequences [25]. Thus, social support plays an important modulating role in the relationship between stress and health [19]. Another element that may help explain the stable evolution of work-related stress and self-rated health during the recession is the theory of life events. Life events are discrete events that occur at certain moments associated with major changes in one’s life [64]. These events may have a positive or a negative influence on health. The economic recession, which began in 2008, was a key life event for all those who lost their job stability as a result of it. In this sense, some studies have shown that the ability to cope correctly with life events is directly related to an individual’s social support [65].

Job satisfaction and work-related stress remained stable during the recession. This finding is consistent with that reported by other authors in the scientific literature. According to Sánchez-Sellero et al. [66], the recession was one of the factors, which had the smallest impact on job satisfaction, with others, such as activity, personal development and motivation explaining this variable better [67]. It is important to remember that job satisfaction is strongly influenced by one’s own feelings about the work environment, meaning that, when faced with situations of job instability, people tend to view as satisfactory certain elements, which they previously viewed in a negative light [68].

Finally, our results reveal how social support acted as a positive mediator of the relationship between work-related stress and self-rated health only in 2006, prior to the onset of the recession, whereas later on (2001–2017), this mediating role was not observed. This is consistent with that described previously in the scientific literature. We live in an individualistic, digitised society in which digital relationships take precedence over personal ones. Consequently, we are now faced with what some have started to call the “epidemic of solitude”. Studies have shown that good social networks are instrumental in helping people cope with and overcome situations of recession and job instability and loss. However, our results also show that only apply to men, younger workers, those with qualified jobs and those with a high education, but not to women, older workers, those with unqualified jobs, or low education. These data align with the Conservation of Resources Theory [69] that posits that people make many efforts to maintain, accumulate, and build resources (objects, personal or psychological characteristics, conditions such as money or knowledge), and that a perceived threat of losing (e.g., job insecurity, losing one’s job) resources can lead to feelings of distress and reduced well-being. These perceived threats (or real situation) would be even harder for marginalised groups, such as women, older employees, or low-income families or immigrants [69,70], because they could lack the resources such as social support or resilience (key factors to preserve mental health) [71,72] to offset loss. This situation could place women, older workers, and those with unqualified jobs or low education in an even greater resource loss spiral relative to men or (young) workers with qualified jobs and higher education and, therefore, be more vulnerable to additional losses [73].

This study has certain limitations, which should be taken into account. The first is linked to the information source used, namely the NHSs. Few studies have used this instrument to study the occupational health of the population, although the few that have found it to be both useful and reliable. Another limitation is the way in which the NHSs measure stress and job satisfaction, particularly since there are currently numerous validated scales that facilitate the analysis of these variables and their different components. Despite this, however, the results returned by this instrument for stress and job satisfaction coincide with those found using other instruments that have been validated for the Spanish population. Finally, it is important to highlight those limitations inherent to cross-sectional studies, the results of which cannot be used to infer causality, although they do serve to draw conclusions. Despite these limitations, however, the NHSs are surveys that are administered repeatedly to the same population at different moments in time, providing a (fairly) faithful picture of the situation being studied. Consequently, we believe that the present study is relevant. The extant literature contains very few studies like this that analyse the relationship between work-related stress and self-rated health in the population, and the mediating role played in that relationship by perceived social support and job satisfaction during economic recessions.

## 5. Conclusions

The 2008 financial crisis and subsequent recession had a strong impact on employment, rapidly increasing unemployment, especially in fields linked to the “real-estate” bubble. In different European countries, and Spain in particular, the lack of social and employment policies further exacerbated the situation, hampering their ability to cope and respond adequately. Despite this, however, the population who remained in employment has shown a high level of resilience and a good capacity to adapt to the new circumstances, as reflected in the fact that certain indicators, such as work-related stress and occupational health, did not drop. The current health crisis caused by the COVID-19 pandemic has triggered a new recession, which may well be even deeper than the previous one. Job instability, insecurity and loss have all risen in a much shorter space of time than in 2008. Studies such as this one help us to understand how the working population may react to the new situation. Moreover, past lessons may help improve the way we cope with future difficulties. We need active social and employment policies to help both the working population and society in general cope adequately with situations of financial crisis and recession. We also need more long-term studies to observe the impact of these circumstances on workers’ health. Finally, we should not overlook the importance of analysing the effect of intermediate indicators, such as the use of health services by the working population faced with situations of ongoing stress.

## 6. Practical Implications

The National Health Surveys provide periodic information, which enable different determinants of health to be studied in the Spanish population. These determinants include work-related stress, self-rated health, social support and job satisfaction, which is why, despite their possible limitations, the NHSs are a useful tool for monitoring and assessing intervention strategies from both an occupational and health-related perspective. In relation to the data reported here, throughout the entire study period, job satisfaction was found to positively mediate the relationship between work-related stress and self-rated health. Human resource management practices should be aimed in two main directions. First, from a preventing perspective, they should be focused on reducing work stress, both by organizational interventions and by individual strategies. As recent developments showed [17], organizations would reduce work-related stress by increasing job resources and reducing hindering job demands. At the individual level, each employee could intervene by job crafting strategies [67,74], that can help to increase the adjustment of job demands to his or her abilities and skills. Second, organizations also could try to promote job satisfaction, given its buffering role into the relationships between stress and health. Moreover, high job satisfaction levels have also been linked to greater productivity and lower rates of absenteeism. According to the Job Demands and Resources model [39,41], increasing challenging demands would improve employee’s satisfaction, followed by providing them with useful resources to cope with these demands.

The present study also reveals how Spanish society is becoming increasingly individualistic. Nevertheless, social support was found to have a positive effect on the relationship between work-related stress and self-rated health among the working population. Loneliness has been described as the great epidemic of the 21st century. This study points to the need for social and education policies that fight against this “loneliness”, in order to ensure a more socially active population. As the Social Cure Theory stated [75], multiple group membership could be considered a protective factor to cope with disease and affliction, based on recent empirical findings [76]. Finally, the study also serves to highlight the consequences of the 2008 financial crisis and subsequent recession on stress, job satisfaction and perceived social support among the working population of Spain, and how these variables influence self-rated health.

This information should serve as a guide to react to the current recession caused by the COVID-19 pandemic. This information should serve as a guide to react to the current recession caused by the COVID-19 pandemic. In order to increase jobs and curb unemployment, at the political level, the quality of jobs, and therefore the health of workers, can be neglected. It is important to generate employment, but in healthy conditions, where we are able to articulate exposure to new professional risks, such as current biological risks, with greater socialization of workers, stress reduction, and satisfaction. Occupational health services must pay attention to these new circumstances and to the new challenge of trying to protect the health of workers in times when less should achieve more.

## Figures and Tables

**Table 1:** Description of the sociodemographic and health (mental health) characteristics of the working population. Disaggregated by sex. Spain (2006–2017).

	Men (%)				Women (%)			
	2006	2011	2017	*p*(χ^2^)	2006	2011	2017	*p*(χ^2^)
	(*n =* 6579)	(*n* = 4982)	(*n* = 5222)		(*n* = 6490)	(*n* = 3998)	(*n* = 4697)	
Sociodemographic Variables								
Social class *	%	%	%		%	%	%	
I	11.3	14.5	13.2	0.241	12.6	13.6	15.1	0.103
II	11.5	8.5	9.3	0.116	13.1	10.2	10.9	0.141
III	25.1	19.8	20.5	0.016	26.2	22.7	22.4	0.052
IV	18.5	16.7	15.7	0.098	20.6	11.6	9.5	<0.001
B	12.1	29.8	30.9	<0.001	15.1	26.1	28.5	<0.001
VI	10.9	9.9	10.1	0.226	11.3	15.1	13.1	0.283
Education level **								
High	30.7	59.3	37.2	0.024	37.4	39.1	47.1	<0.001
Medium	38.9	31.4	51.2	<0.001	37.3	53.5	44.7	<0.001
Low	29.9	9.3	11.5	<0.001	25.2	7.5	8.1	<0.001
Health Variables	*M (SD) ^1^*	*M (SD) ^1^*	*M (SD) ^1^*	*p* (Student’s *T*)	*M (SD) ^1^*	*M (SD) ^1^*	*M (SD) ^1^*	*p* (Student’s *T*)
Mental Health ***	4.8 (0.2)	3.2 (0.08)	9.1 (0.05)	<0.001	4.7 (0.21)	3.1 (0.07)	9.8 (0.06)	<0.001

Note. * Social class: I: Directors and managers with university degrees; II: Directors and managers with less than 10 workers, professions linked to diplomas; III: middle management and self-employed workers; IV: supervisors and workers in jobs requiring technical qualifications; V: qualified workers in the primary sector and other semi-qualified jobs; and VI: unqualified jobs. ** Education level: High: those with university degrees and/or advanced vocational training; Medium: those with secondary level qualifications and/or mid-level vocational training; and Low: those with primary qualifications or no studies. *** Mental Health: scores from 0 to 12, indicating better to worse mental health. 1. M(SD): mean and standard deviation.

**Table 2:** Descriptive statistics and correlations of the criterion: self-rated health; predictor: work-related stress; and mediating variables: job satisfaction and perceived social support.

	2006				2011				2017			
Variables	*M (SD)*	2. S	3. JS	4. SS	*M (SD)*	2. S	3. JS	4. SS	*M (SD)*	2. S	3. JS	4. SS
1. Self-rated Health ^1^	3.9 (0.7)	−0.074 **	0.11 **	0.06 **	4.0 (0.7)	−0.08 **	0.13 **	0.08 **	4.0 (0.8)	−0.09 **	0.14 **	0.16 **
2. Work-related Stress ^2^	4.2 (1.7)		−0.11 **	−0.007	4.3 (1.7)		−0.10 **	−0.01	4.3 (1.7)		−0.12 **	−0.28 **
3. Job Satisfaction ^3^	5.1 (1.5)			0.06 **	5.5 (1.4)			0.09 **	5.5 (1.4)			0.16 **
4. Perceived Social Support ^4^	51.1 (12.4)				50.0 (10.2)				43.6 (6.3)			

Note.^1^ Self-rated health ranges from 1 (poor) to 5 (very good). ^2^ Work-related stress ranges from 1 (very low) to 7 (very high). ^3^ Job satisfaction ranges from 1 (very low) to 7 (very high). ^4^ Perceived social support ranges from 11 (very low) to 55 (very high). ** *p* < 0.001.

## Data Availability

The data used for this study are public and accessible to anyone through the links provided by the Spanish Ministry of Health, Social Services and Equality (https://www.mscbs.gob.es/estadEstudios/estadisticas/encuestaNacional/, accessed on 30 March 2021).
